# Supplementary material for: Distinct mesoderm migration phenotypes in extra-embryonic and embryonic regions of the early mouse embryo
Source: eLife. 2019 Apr 5;8:e42434. doi: 10.7554/eLife.42434 (PMC6450669; doi:10.7554/eLife.42434)
Supplement: Transparent reporting form [file elife-42434-transrepform.docx]

***eLife’s* transparent reporting form**

We encourage authors to provide detailed information *within their submission* to facilitate the interpretation and replication of experiments. Authors can upload supporting documentation to indicate the use of appropriate reporting guidelines for health-related research (see [EQUATOR Network](http://www.equator-network.org/%20)), life science research (see the [BioSharing Information Resource](https://biosharing.org/)), or the [ARRIVE guidelines](http://www.plosbiology.org/article/info:doi/10.1371/journal.pbio.1000412) for reporting work involving animal research. Where applicable, authors should refer to any relevant reporting standards documents in this form.

If you have any questions, please consult our Journal Policies and/or contact us: [editorial@elifesciences.org](mailto:editorial@elifesciences.org).

**Sample-size estimation**

- You should state whether an appropriate sample size was computed when the study was being designed
- You should state the statistical method of sample size computation and any required assumptions
- If no explicit power analysis was used, you should describe how you decided what sample (replicate) size (number) to use

Please outline where this information can be found within the submission (e.g., sections or figure legends), or explain why this information doesn’t apply to your submission:

All sample size information is found within the corresponding figure legend, except for “Figure 6-Supplementary Figure 1” for which it can be found within the result section entitled “Extra-embryonic mesoderm migration is Rho GTPases independent”.

For the quantitative analysis of cell morphology and movement in wild-type embryos, there was no *a prio*ri when the study was initiated. The differences described were first observed qualitatively, and then quantified on live imaging data from representative embryos. The results obtained were highly statistically significant, and confirmed the qualitative assessment.

For mutant embryos analysis, there was again no a priori hypothesis that would have allowed power analysis. We included all mutant embryos we obtained in the analysis, and present the data detailing the frequency of phenotypes.

**Replicates**

- You should report how often each experiment was performed
- You should include a definition of biological versus technical replication
- The data obtained should be provided and sufficient information should be provided to indicate the number of independent biological and/or technical replicates
- If you encountered any outliers, you should describe how these were handled
- Criteria for exclusion/inclusion of data should be clearly stated
- High-throughput sequence data should be uploaded before submission, with a private link for reviewers provided (these are available from both GEO and ArrayExpress)

Please outline where this information can be found within the submission (e.g., sections or figure legends), or explain why this information doesn’t apply to your submission:

All information relative to the number of experiments is included either in the figures/tables legends or in the text. Only biological replicates were performed, as each experiment is performed on a different embryo or set of embryos (for RNASeq, cf Figure 4_Source Data 1).

We did not exclude any data. For qualitative and quantitative analysis of wild-type embryos, embryos were chosen according to the technical quality of imaging.

**Statistical reporting**

- Statistical analysis methods should be described and justified
- Raw data should be presented in figures whenever informative to do so (typically when N per group is less than 10)
- For each experiment, you should identify the statistical tests used, exact values of N, definitions of center, methods of multiple test correction, and dispersion and precision measures (e.g., mean, median, SD, SEM, confidence intervals; and, for the major substantive results, a measure of effect size (e.g., Pearson's r, Cohen's d)
- Report exact p-values wherever possible alongside the summary statistics and 95% confidence intervals. These should be reported for all key questions and not only when the p-value is less than 0.05.

Please outline where this information can be found within the submission (e.g., sections or figure legends), or explain why this information doesn’t apply to your submission:

Statistical methods information is found in the figures and tables legends where applicable. We used the t test when the distribution was Gaussian with equal variances. For non-parametric distribution we used the Mann–Whitney–Wilcoxon.

(For large datasets, or papers with a very large number of statistical tests, you may upload a single table file with tests, Ns, etc., with reference to sections in the manuscript.)

**Group allocation**

- Indicate how samples were allocated into experimental groups (in the case of clinical studies, please specify allocation to treatment method); if randomization was used, please also state if restricted randomization was applied
- Indicate if masking was used during group allocation, data collection and/or data analysis

Please outline where this information can be found within the submission (e.g., sections or figure legends), or explain why this information doesn’t apply to your submission:

Not Applicable.

**Additional data files (“source data”)**

- We encourage you to upload relevant additional data files, such as numerical data that are represented as a graph in a figure, or as a summary table
- Where provided, these should be in the most useful format, and they can be uploaded as “Source data” files linked to a main figure or table
- Include model definition files including the full list of parameters used
- Include code used for data analysis (e.g., R, MatLab)
- Avoid stating that data files are “available upon request”

Please indicate the figures or tables for which source data files have been provided:

The quantification data for Figure 2 and Table 1 and 2 are found in Figure 2_Source Data 1, 2 and 3, and for Figure 1- Supplementary Figure 1 in Figure 1- Supplementary Figure 1_Source Data 1. Data for Figure 3 are in Figure 3_Source Data 1. Data related to cell division, cells in proximity, and cell collision (described in main text) are in Figure 3_ Source Data 2, 3, and 4, respectively.

RNA Seq source data (Figure 4 and Figure 4-Supplementary Figure 1) have been uploaded on https://datadryad.org/. In addition, read counts in LogFPKM and differential expression analyses are found in Figure 4_Source Data 2 and 3. The quantification data for Figure 6-Supplementary Figure 1 are in Figure 6-Supplementary Figure 1_Source Data 1 and 2, and for Figure 6-Supplementary Figure 2 in Figure 6-Supplementary Figure 2_Source Data 1.
